# Blueprint and Implementation of Rural Stand-Alone Power Grids with Second-Life Lithium Ion Vehicle Traction Battery Systems for Resilient Energy Supply of Tropical or Remote Regions

**DOI:** 10.3390/ma12162642

**Published:** 2019-08-20

**Authors:** Antonio Nedjalkov, Jan Meyer, Heiko Göken, Maximilian V. Reimer, Wolfgang Schade

**Affiliations:** 1 EST Research Center Energy Storage Technologies, Clausthal University of Technology, Am Stollen 19A, 38640 Goslar, Germany; 2 Department for Fiber Optical Sensor Systems, Fraunhofer Heinrich Hertz Institute, Am Stollen 19H, 38640 Goslar, Germany; 3 Institute of Management and Economics, Clausthal University of Technology, Julius-Albert-Straße 2, 38678 Clausthal-Zellerfeld, Germany

**Keywords:** rural power supply, off-grid network, second-life battery, lithium ion, electromobility, stationary energy storage, fiber-optical status monitoring, fiber Bragg grating, economic development, key decision variables

## Abstract

Developed societies with advanced economic performance are undoubtedly coupled with the availability of electrical energy. Whilst industrialized nations already started to decrease associated carbon emissions in many business sectors, e.g., by substituting combustion engines with battery-powered vehicles, less developed countries still lack broad coverage of reliable electricity supply, particularly in rural regions. Progressive electrification leads to a need for storage capacity and thus to increasing availability of advanced battery systems. To achieve a high degree of sustainability, re-used batteries from the electromobility sector are appropriate, as they do not consume further primary resources and still have sufficient residual capacity for stationary electrical storage applications. In this article, a blueprint for the electrification of a remote region by utilizing second-life lithium ion traction batteries for an integrated energy system in a stand-alone grid is presented and the implementation by the example case of a Tanzanian island in Lake Victoria is demonstrated. First, economic potentials and expected trends in the disposability of second-life lithium ion batteries and their foreseeable costs are outlined. Subsequently, key decision variables are identified to evaluate logistic aspects and the feasibility of the implementation of an off-grid electrical system in remote areas for economically and geographically unfavorable environments. The practical realization is pictured in detail with a focus on technical performance and safety specificities associated with second-life applications. Therefore, a new type of battery management system is introduced, which meets the special requirements of climate compatibility, low maintenance, enhanced cell balancing capability and cell configuration flexibility, and combined with a fiber-optical sensor system, provides reliable status monitoring of the battery. By carrying out on-site measurements, the overall system efficiency is evaluated along with a sustainability analysis. Finally, the socioeconomic and humanitarian impact for the people on the island is debated.

## 1. Introduction

Great efforts are being made in all parts of the world to replace fossil fuels for power generation and in the transportation sector by renewable energy sources. The storage of electrical energy plays a central role in this global endeavor. Batteries are evolving into a key component capable of fulfilling the associated requirements. Notably, the automotive industry has become one of the major drivers of innovation in the battery manufacturing segment over the past five years. Currently, the predominant technology is lithium ion batteries. No other type of commercial energy storage reaches comparable energy densities with a concomitant high-performance and cyclization stability. However, the major disadvantages of the lithium ion battery include high purchase price, uncertain availability of rare raw materials required for production and unclear recycling potential. Consequently, it is expedient and sustainable to use a battery storage of this technology over a prolonged lifespan before disposal. In particular, electric vehicle applications place high demands on the traction battery. After the end of the life cycle, which is usually specified at a state of health of 80%, these components still provide enough residual electrical capacity to be transferred into a further application. Practically each sold electric vehicle leads to a return flow of a traction battery, which turns its respective manufacturer or owner into a potential supplier of energy storage devices or pre-used cells. Accordingly, applications with less stringent requirements for performance and gravimetric energy density are demanded, which, however, have a clear benefit for technological advantages and are likewise sensitive in terms of the purchase price. Stationary energy storage systems are particularly appropriate for the utilization of second-life lithium ion batteries, because they supply and store electricity as needed or provide additional power for the local grid, for example for fast-charging stations of electric vehicles or frequency control tasks, without strict requirements on volume and weight specifications.

A specific scenario of developmental interest emerges for stand-alone power grids that are separated from the national grid and exist where a deliberate disconnection is preferred because self-production is more cost-efficient or where no connection is economically or technically feasible for the geography under consideration, as it is often applicable in tropical or remote regions. Operators of these stand-alone grid networks are thus potential customers for second-life battery storages. Lithium ion batteries are well suited for rural power supply due to their cycle stability without a significant loss of capacity over a long service life and the accompanying low maintenance effort. However, it must be taken into account that even brand-new lithium ion cells, as a result of their high specific energy density, pose a major hazard potential, which can result in devastating accidents in the event of a malfunction or during an operation outside of the specification limits. For this reason, the monitoring of the operating conditions is of central importance when batteries of this cell chemistry are applied. To accomplish this, a battery management system is deployed, which records the state variables voltage, current and temperature and adjusts the operating mode based on the condition analyses and predefined system parameters. As even tougher safety requirements apply to second-life batteries, since degradation processes that have already occurred during the first life cycle are often unknown, a battery management system with specific attributes and an operating mode that can be individually tailored to the intended purpose is indispensable. In addition to the integrability of supplementary sensing technologies, such as fiber-optical measuring systems for a redundant determination of the state of charge and state of health, furthermore, an intelligent malfunction detection and the capability to combine different cell classifications and types are essential, making the battery management system the key component for a safe and efficient operation for pre-used, and if necessary, refurbished battery storages.

The implementation of energy storage systems in an off-grid operation mode in remote regions requires, in general, a high planning and development effort due to many unknown accompanying parameters and the prevailing system individuality. Moreover, although the realization of such projects is technologically feasible, in many cases it cannot be financed by the users itself. Plant sites also perchance are located in hardly accessible areas with potential safety concerns. However even if the assembly and operation of energy systems in rural areas may not be economically viable without supporting subsidies, they, in turn, constitute an enormous development potential for the regions concerned and represent major humanitarian progress, above all, through the support for the healthcare and education sectors. In view of this fact, the aspiration of this article is to present a comprehensive approach for the construction and subsequent realization of stand-alone power grids with second-life lithium ion batteries as energy storages in tropical and remote regions, using the concrete example of the island of Kibumba in the southwestern Lake Victoria in Tanzania. In the following Section 2, initially, the technological and economic potential for the utilization of second-life batteries is assessed with a focus on vehicle traction batteries as the resource. Subsequently, in Section 3, the necessary prerequisites and environmental parameters for the implementation of energy storage devices in remote stand-alone grids are described and key decision variables for the evaluation for potential electrification of suitable areas are presented. Based on the example of the island of Kibumba, in Section 4, it is demonstrated how a project development and initiation can be carried out under unfavorable environmental conditions, which main components have to be designed and constructed for the energy system and which expenses as well as effort must be expected for transport and installation. By means of a high-load charging and discharging experiment, which is presented in Section 5, the suitability and functionality of the applied individual components are validated by an analysis of the solar, electrical and temperature monitoring data. Conclusively, in Section 6, the assembled system is placed in a socioeconomic context and its sustainability critically determined from technological and societal perspectives.

## 2. Potential for Utilization of Second-Life Lithium Ion Batteries

As previously indicated, the automotive industry is a major force in the further development of lithium ion batteries. For the application of vehicle traction, particularly battery cells with a large capacity and gravimetric energy density are appropriate. In the three prevailing structural shapes, pouch cell, prismatic cell and round cell, single-cell capacities up to 96 Ah and an energy density of 280 Wh/kg are achieved [1,2]. Reliable price information for batteries is usually difficult to determine, as these depend substantially on the requested amount and cell chemistry. Typically, for the current generation of lithium ion batteries, an average cost level of 100–200 €/kWh is specified [3,4,5].

In 2016, the total production capacity of this battery storage type was 198 GWh [6]. Thereof, an energy amount of 103 GWh was attributed to traction batteries for the electric vehicle [7]. By 2025, for electric vehicles, a demand of 500–750 GWh and for stationary storage up to 100 GWh [8] is expected with a cost level of approximately 100 €/kWh [7]. In the same year, it is forecasted that an energy amount of 95 GWh, according to the original mint condition, is transferred back to the market through returned cars [9]. A share of 26 GWh is attributed to be subsequently used in stationary storage facilities [9]. Consequently, it is essential that also the price for offered second-life battery storages coincidently drops with that of lithium ion batteries straight from the factory. The current required market price for these types of pre-used storage is given as 88 €/kWh so that a substantial cost advantage of second-life batteries prevails [10]. 

The electric vehicle market is predominated by the USA, Europe and China. The worldwide sales of these vehicles in 2020 are expected to be ten million units, in 2025, 31 million units, and in 2030, 54 million units. Of this total, in 2020, one million units will be allocated to the USA, two million units to Europe and six million units to China, in 2025, five million units to the USA, seven million units to Europe and 14 million units to China, and in 2030, ten million units to the USA, eleven million units to Europe and 23 million units to China [11]. Based on these values, return flow quantities into the market can be calculated, which aim to assess the potential for recycling by deriving returns from past sales. By using given sales data as well as forecasts for future sales, it is customary to assign an average lifespan and retrieve returns analytically or by simulation. Normal and Weibull distributions are only two of the possible density functions, each requiring location parameter assumptions suitable for the returned goods [12]. For the special case of lithium ion battery recycling, the batteries can return before or exactly when the vehicle’s lifetime is reached, de facto creating a unique lifetime distribution [13]. Whether the latter can be approximated without significant error by a single density function, such as one of the abovementioned, depends on the underlying lifetime distributions for cars and batteries, respectively. In consideration of these models, a return volume of more than 5 GWh will be available to the European market in 2025, which can be used for second-life applications [14]. This corresponds to approximately 67,000 stationary energy systems as realized by our group for the present project.

In the implementation of battery storages assembled from pre-aged lithium ion cells, there are a variety of both technical and economic questions. For a sufficient performance of the system, it is desirable to merge battery cells of the same type and uniform degradation level. Consequently, it is essential either to know the event history during the first life cycle or to have the capability to perform rapid tests on the state of health on a single cell level. For the latter topic, there is a whole range of research work addressing, for example, impedance spectroscopy [15], voltage jump responses [16], ultrasonic layer analysis [17] or surface scanning [18]. On the other hand, there is a clear potential for application in permanent single-cell monitoring, for example, by means of electrical [19] or optical surface strain sensors [20] to specifically detect aging effects. Alongside the power storage operation, evaluation points can be assigned by the higher-level battery monitoring unit, which enables analysis and classification at the end of the first life cycle [21]. Decisive for this function is above all the battery management system. This component represents an essential key competence for all battery manufacturers, as it has a substantial effect on the lifetime and safety during operation. Especially for second-life applications, the monitoring system requires a high degree of flexibility, since more advanced balancing must be achieved between the individual cells and the probability of safety-critical conditions increases. As a preliminary work for the project presented here, our group has developed a battery management system specifically designed for second-life lithium ion battery applications, which is used in the built-up energy system [22]. 

Even if pre-aged traction batteries return from their first use, they might be cumbersome to purchase for external third-party storage providers, as the original equipment manufacturers are likely to remain in the possession of the product and will refrain from transferring their technology into inscrutable re-use with security and reputational risks. Accordingly, they will strive to realize their own second-life storage projects autonomously. In fact, major manufacturers have already implemented a number of joint projects and built up the corresponding know-how. In Table 1, examples of stationary energy storages with second-life batteries from the application field of electric vehicles are listed.

Regardless of the aspirations described above, the vast majority of battery systems for stationary applications assembled so far are still based on the established lead-acid battery technology. The regarding cells are comparatively inexpensive to purchase at a price of 100 €/kWh [28] and also stand out by a safety-uncritical operation. The low energy density and the associated high weight per kilowatt hour are of secondary importance for stationary applications. The current generation of lithium ion batteries, on the other hand, requires diligent monitoring because of cell chemistry and extremely high energy density. In addition, the characteristic temperature operating range requires an intensive effort for the system’s temperature control unit. Nevertheless, lithium ion batteries are technologically more suited also for stationary power storage, since the long-term maintenance effort is very low due to the extraordinary cyclization stability and the omitted electrolyte treatment. However, the main determining factor for choosing the most appropriate energy storage is its price. The primary objective of second-life battery developments is thus, by combining a significantly lower price compared to brand-new batteries with technology-related advantages, to create an economic basis for previously not economically feasible project plans for electricity storage. These circumstances are of particular significance if energy systems with second-life battery storages are implemented in remote regions found in developing countries with restricted financial and infrastructural resources. The characteristic requirements are outlined in the next section. Subsequently, key decision variables are introduced and applied to find regions best suited for electrification.

## 3. Remote Regions for Electrification with Stand-Alone Power Grids

### 3.1. Regional Prerequisites and Conditions for Project Initiation

Focused in this work are battery storage systems in off-grid operation without access to the public grid infrastructure. Although such systems have very high electricity generation costs, a stand-alone grid is most likely appropriate in case the installation of the infrastructure in relation to the expected demand is out of balance, for example, if a natural barrier exists or extremely long distances must be overcome. Under preferable environmental conditions, usually combined with a governmental price incentive scheme, a selective decoupling from the power grid by consuming the self-generated electricity can also be economically worthwhile. Tanzania is a country that is particularly interesting for off-grid projects. Especially in the northern regions with Lake Victoria, dense forest areas and mountain ranges, there are many obstacles, which can only be accessed with significant effort. While 65% of the population in Tanzania lives in remote areas, by 2016 only 7% of this group had access to electricity [29]. The government has therefore initiated a series of measures to achieve not only a significantly higher electricity production capacity but also a noticeably higher number of households with power connection. Together with the World Bank, with the aim to annually implement the power supply for 2000 villages in rural areas with on-grid or off-grid solutions, the “Rural Electrification Expansion Project”, with a term until 2022 and a financing volume of 184 million €, was started [30]. In 2017, the European Union launched a complementary program with a total budget of 44 million € to provide access to electricity for 3600 villages [31]. The comparatively good investment climate and the foreign willingness to commit funding are favored by the relatively stable political and social conditions in the country, which create security for continuity in the intended development efforts.

For the construction of an off-grid power network, essentially three major components are needed. In addition to the actual electrical cable infrastructure between the supplier and the consumer, a power generator, a network converter and an energy storage device are required. Without a connection to the main power grid, solar energy is the most appropriate source of electricity in Tanzania’s climate. Also conceivable and widespread are diesel generators. The supply of fuel, however, is a problem, particularly for remote regions. Besides, this type of production causes local noise and exhaust emissions. For this reason, it is desirable to use diesel generators only to bridge electricity shortages. The power converter, in accordance with the grid type in a single-phase or three-phase design, regulates the current flows in the system and generates the alternating current for the power grid. DC power grids are also feasible, but only for short transmission paths in the regarding voltage range. In addition, their operation leads to difficulties in the use of commercial electrical equipment. Since generation and consumption, especially in off-grid networks, must correlate at any time, it is necessary to integrate a power storage unit. As described before, lithium ion batteries are particularly suitable for this purpose, if technological and economic hurdles can be overcome. In the following, it is demonstrated which parameters within a geographical balance limit are applied in order to evaluate decoupled regions according to their potentials for electrical infrastructure development.

### 3.2. Key Decision Variables for the Evaluation and Selection of Considered Regions

Due to the tremendous divide in infrastructural development between densely populated and remote areas, in Tanzania specifically, places that are far or isolated from major cities have the potential for electrification with rural power supply. This involves primarily islands and some of the areas in the mainland blocked by a natural barrier. Due to limited budget and expert resources, the national supply utility company focuses on highly populated areas closer to the existing grid. In contrast, rural regions are not as profitable for a connection to the national power network [32,33]. 

Electrification can be economically appropriate through independent initiatives if external funding or technical equipment are provided and supported by a specialized organization for rural energy supply. Whether projects are realized or not is evaluated by means of key decision variables, which are defined by these organizations. In general, they assign higher priority to commercial activities over individual demands. Accordingly, an assessment is initially conducted to identify the ratio between private and business development opportunities. Another factor is the number of inhabitants or the number of people who live temporarily or permanently in the region concerned as well as the expected population growth. Furthermore, there is an analysis of the people’s daily activities like employment status, economic performance or commuting. The decision is also influenced by the number of houses and a determination of their condition. Likewise, the distribution of houses is implemented. A large spread of houses is accompanying with an increased material consumption for electrical exploitation, which has an adverse effect on the decision. From a technical point of view, primarily, the energy system’s expected lifespan, operational reliability, maintenance effort, safety and installation costs as well as the technology-specific funding eligibility are decisive. If the rural power supply organization evaluates projects based on the aforementioned aspects exclusively, soft factors such as nature conservation beyond legal requirements, development aid and knowledge from test facilities in realistic environmental conditions as well as humanitarian aspects like medical and educational development can be undervalued.

In case of a decision in favor of an electrification project, exclusive contracts are concluded with the local authorities for most commonly 30 years, which limit the activities of other investors in the area concerned. An exemplary player in the field of specialization for electrification and power supply in remote regions in Tanzania is Jumeme Ltd., which also acted as a partner for the project presented here. Apart from this, the organization is currently implementing 14 individual projects. Among them are ten villages in the Kigoma region alongside Lake Tanganyika, which will be electrically connected to a stand-alone grid as the national power network is not available. The greatest development potential is found in the southern regions of Lake Victoria. There is a vast number of small but inhabited islands without power connection. Jumeme Ltd. applied the aforementioned economical specifications and four of the islands are now in the process of electrification with a stand-alone grid. Due to the lack of technological resources, such as monitoring electronics and operating knowledge, all of these systems will be equipped with lead-acid batteries for energy storage. As a result of the cooperation, a technology transfer is possible that represents a clear economic advantage in the business environment of the organization. In the following section, the key decision variables are applied to the concrete example of Kibumba Island.

## 4. Pilot Project: Kibumba Island

### 4.1. Initial Situation and Project Goals

The archipelago Nazinga in Lake Victoria consists of eight individual islands. Thereof, two were positively evaluated due to their high population and economic activity in the fish processing industry. In contrast, the island of Kibumba (coordinates: 2°01’ S, 31°47’ E), addressed in this article, was given a negative rating because of the comparatively small number of 150 inhabitants and the lack of production. In terms of business activity, only a store, a food stand, a bar and fishery exist. Aggravating the initial situation, the housing arrangement is exceedingly sprawled, which leads to correspondingly complex infrastructure construction.

Despite these disadvantages, Kibumba has a significant humanitarian impact on the whole archipelago. According to the Ujamaa society model, each village community was obligated to organize self-sufficiently with a minimum use of resources. Consequently, only one school and one infirmary in this region exist, which are both located on the centrally positioned Kibumba Island and are a focal point for all residents of the Nazinga archipelago. An exclusion of electrical power for these vital facilities would be a significant barrier for the development opportunities of the entire region. By including these humanitarian factors in the key decision variables, electrification of Kibumba proved to be most impactful. With Jumeme Ltd. it had been agreed that, without any share of expenses, an energy system based on second-life lithium ion batteries would be built on the island as a pilot plant by our group. In return, the partner obtains the complete system and manages the installation of power cables between consumers, subsequent system maintenance and any customer care.

After extending the key decision variables resulting in a favorable evaluation for electrification of the island Kibumba, first, the development of the electricity demand was analyzed for the following five years. The focus was on the two before mentioned objects, school and infirmary, as well as on the existing fishing lodge, that each was electrically implemented in parallel to the energy system. In order to provide enhanced access to education and facilitate administrative work, the school has been provided with power cables, sockets and lighting by means of an associated charity project. In this context, computers and printers were also procured. In a similar way, this was realized for the infirmary. By equipping the building with an electrical connection, a cooling system for medicines has already been set up and in the future, it will be possible to operate electrical health screening equipment. The third focus was on the fishery, the only economic sector in the region. Through electrification and the organized ice cube machine, it is now possible to freeze the fish catch and transport it without interruption of the cold chain to the markets on the mainland. Since the electricity offered is very expensive in relation to the average income of a private person, the connection to ordinary households is expected with considerable delay and is therefore subordinate to the main project objectives. The price structure for the electricity is staggered into three parts. For public institutions, like the infirmary and the school, as well as private households the tariff is 1.35 €/kWh. In contrast, a private individual has an estimated buying power of 1–1.5 € per day. Commercial customers, like shops and other small businesses, have to pay 0.96 €/kWh and for manufacturing facilities with machinery, such as mills or processing plants, the price level is 0.29 €/kWh with an additional weekly connection fee of 3.83 €. However, the latter group is not represented on Kibumba. Based on the estimated customer potential, a maximum requested power supply capacity of 3.125 kW and maximum daily energy demand of 18.75 kWh were assumed. In accordance with this presupposition, the individual main technical components were designed or assembled as described in the next section.

### 4.2. Main System Components and Configuration

#### 4.2.1. Off-Grid Converter

**Figure 1:**
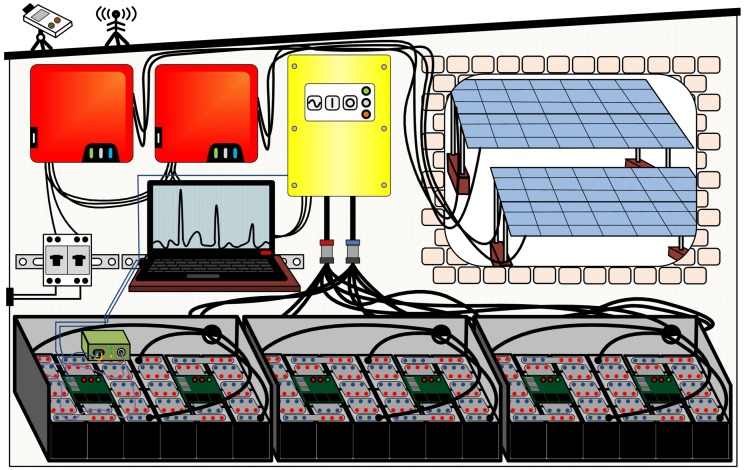
Schematic representation of the energy system structure; each of the six battery cell strings is monitored by a battery management system. One string is additionally equipped with fiber-optical sensors. The battery is connected on the DC side to the off-grid converter. The DC produced by the photovoltaic system is transformed by the two inverters, which frequency-controlled feed the power grid generated by the off-grid converter.

To simplify the traceability of the operating principle, the structure and operation of the entire energy system are graphically schematized in Figure 1. A brick house with a floor area of 2 m × 3 m and a height of 2 m was built to accommodate the weather-sensitive devices. The regulating centerpiece of the assembling is the off-grid converter. For the expected requirements, a single-phase 50 Hz and 230 V energy system is sufficient. Selected was a Sunny Island 8.0H (SMA, Niestetal, Germany, yellow device in the center of Figure 1) with a rated power of 6.0 kW and a rated AC of 26 A. The rated input DC is 136 A and the rated output DC is 115 A. The system is designed in such a way that the latter values, which in operation represent the discharge and charge current of the battery, are the limiting performance parameters, which correspond, with a rated DC voltage of 48 V, to a power of approximately 6.5 kW and 5.5 kW, respectively.

#### 4.2.2. Photovoltaic Installation

For electricity production, a photovoltaic system was selected exclusively. Pre-owned modules (230P6S, DST, Taipei, Taiwan) with a residual capacity of approximately 90% were therefore utilized. Compared to the brand-new condition, the efficiency had dropped from the original 15% to 13.5%. Taking into account the condition of pre-aging and an estimated total internal system efficiency of 90%, electric power of 6.8 kW must be available for a maximum charging power of the off-grid converter of 5.5 kW. During preliminary measurements at the site, it had become apparent that on average two-thirds of the maximum location-specific solar energy was available due to weather phenomena. Thus, a solar system with a maximum power output of 10.2 kW was required, which corresponded for the respective modules to a number of 44 units with a total module area of 71.71 m^2^ and an effective active material area of 64.25 m^2^. Divided into four parallel strings, the modules were connected in series, resulting in an average string working voltage of 328 V. The orientation of the plant was made in a southerly direction at an inclination angle of 5°.

#### 4.2.3. DC/AC Inverter

In order to convert the DC supply for the grid into AC power, two inverters (Sunny Boy SB5.0, SMA, Niestetal, Germany), each with two connected photovoltaic strings, were integrated into the system (two red devices in the upper left edge of Figure 1). The inverters have a rated feed-in capacity of 5.0 kW, respectively. The off-grid converter controls the feeding behavior of the two inverters by adjusting the grid frequency and thus controls the energy flow in the grid according to the momentary electricity consumption and available storage capacity.

#### 4.2.4. Battery Storage

**Figure A1:**
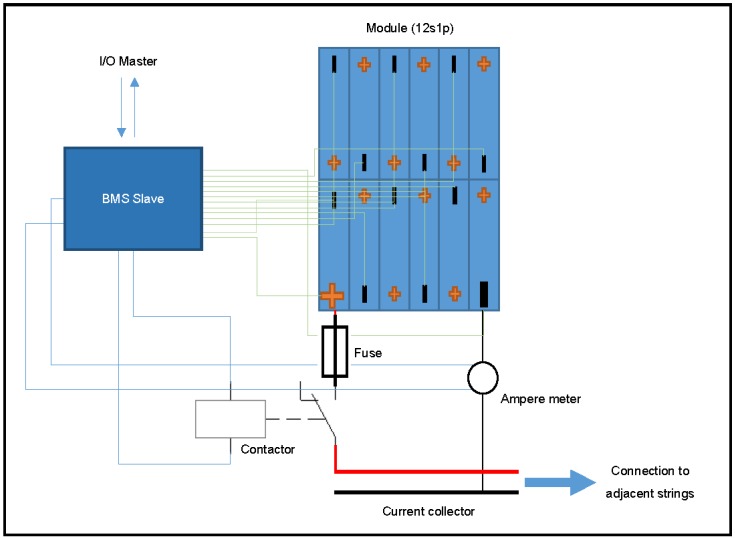
Basic module configuration of one individual string within the assembled battery storage.

The excess energy was stored in the battery and released from it again as required. The battery is the key feature of the development process for the project implementation, and according to the research content, was assembled out of lithium ion battery cells (LMP, Kolibri, Berlin, Germany), which have already been used in a previous application in the field of vehicle traction, in the specific case for trucks for the autonomous transport of shipping containers. Each cell has a capacity of 320 Ah at a weight of 9.3 kg and a normal voltage of 3.7 V. The rated charge and discharge current is 160 A. The entire battery of one vehicle, consisting of 192 cells, was completely disassembled to the cell level initially and subjected to intensive status condition testing. At the time of the examination, the battery had an age of four years. Almost all of the cells had a residual capacity of 93%, which was a very high value for a second-life application, as the automotive industry typically assumes a residual value of 80% for the end of the first life cycle. In the solar pre-measurements on site, the average daily energy input was 5.08 kWh/m^2^. The battery storage was designed to have twice the capacity of the daily energy potential. Considering the available efficient photovoltaic area of 64.25 m^2^ and the efficiency of the modules of 13.5% and the internal energy system of 90%, the energy to be stored was 79.31 kWh. Due to the voltage working range of the off-grid converter, a nominal battery voltage of 44.4 V was applied, which corresponded to a number of twelve cells connected in series. Taking into account the determined capacity loss on pre-aging, this results in an electrical capacity of 13.21 kWh for one string. Accordingly, six equal strings were connected in parallel to attain the required total capacity. The achieved capacity of the battery storage was 79.28 kWh, compared to theoretically 85.25 kWh for cells in mint condition. In order to be able to transport and operate the battery under the later prevailing climatic conditions, for every two strings, sealable metal housings with a dimension of 874 mm × 843 mm were used. An accompanying illustration of the basic battery module configuration of one individual string can be found in Figure A1 of the Appendix A.

#### 4.2.5. Battery Management System

The battery was regulated and main-monitored by the battery management system. For the operation of second-life battery storages, special requirements are involved, which result from the different degree of cell aging and the increased risk of malfunction. In addition, in the present case, difficult climatic conditions and the isolated location of the system with the obligation of a maintenance-free operation were aggravating factors. For these reasons, a completely novel and innovative battery management system, with key features that were presented in the following, was developed specifically for the mentioned demands. In principle, the management system was configured in a master/slave structure. Each of the six battery strings was equipped with a slave system and the first string was additionally provided with the master unit, which gathered all status data as well as communicated via Ethernet with the off-grid converter and a central measuring computer and also switched the main relay. Basically, any master/slave configuration could be realized, so that necessary flexibility was given to build second-life batteries of different energy and performance classes with a matched serial and parallel circuit topology. Due to the increased safety requirement, a three-level protection system was implemented. First, the slaves were verified by the master through a permanent information query function with an embedded logical calculation task. Furthermore, regardless of its own condition after a malfunction, each slave could be put into a safe state from the previous one by switching off the string relay control current, resulting in an open string circuit; the switched-off relay thus defined the safe state. Third, all integrated circuits were provided with a watchdog timer, so that a program error of a device led to an auto reboot, independent of external control units. 

Another special development feature was that the management system had a very high cell balancing capability of 1.08 A per cell, which corresponded to a total balancing power in each string of 55 W at a maximum cell voltage of 4.2 V. Due to the usage of pre-aged cells with potentially greatly varying residual capacities and the very high single cell capacity of 320 Ah, this high power requirement was placed on the balancing system. Under the terms of a maintenance-free system with as few electronic components as possible, a passive balancing unit with ceramic load resistors with a component-specific maximum power per cell of theoretical 7 W was implemented. In order to meet the climatic conditions and extend their lifetime, these parts were equipped with active cooling. In operation, the slaves measured the minimum and maximum voltage values and recognized differences in the state of charge and state of health of cells within a string and compensated them during standby mode by the balancing procedure. For the state calculations, the battery management system used an intelligent and pre-implemented charging detection, so that no external information or supply sources were needed and thus an autonomous operation was enabled.

In addition to the differently implementable master/slave configurations, the free programmability was a decisive target for the development. Therefore, the prerequisites were created to integrate and combine cells of different types and reaction chemistries as well as to implement new analysis algorithms and safety systems. These included, as a complement to the standard monitoring values current, voltage and temperature on a cell as well as string level, for example, fiber-optical sensors, which detect, irrespective of electrical and magnetic interferences, measurement data such as cell expansion, pressures and temperature fields. Moreover, system behavior could be influenced elementarily by the free configuration of trigger values.

**Figure A2:**
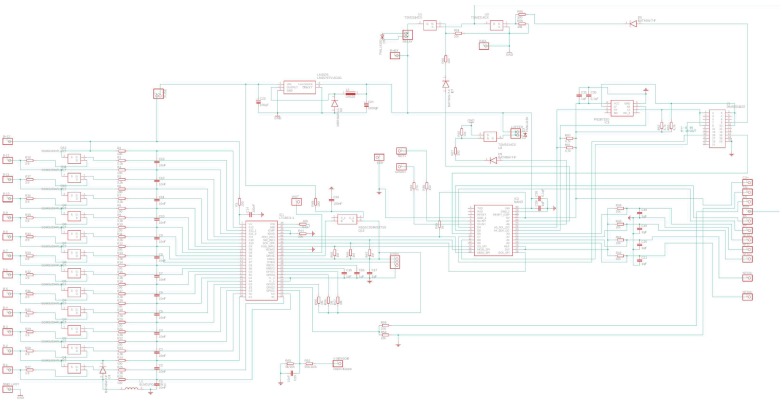
Electrical circuit diagram of the battery management system developed within the framework of this project.

Due to the unfavorable accessibility of the plant and lack of broadband network, data storage and adapted transmission were of crucial importance. The master unit was provided with a memory card that could store internal data at a general memory clock rate of, for instance, 60 s over several decades. In each of the six battery strings, the twelve single cell voltages, with an absolute measurement error of 1.2 mV, as well as the total string voltage, with a total accuracy of <0.1 V, were recorded. Besides, the string current, with an accuracy of 0.1 A, and four temperature points, using thermistor sensors with a resolution of 0.1 K, were monitored. The temperature working range was designed for the widest possible field of application and was between −40 °C and +85 °C. The value query could be performed up to 20 Hz, depending on the desired resolution. At the highest possible resolution of all measured values, the clock rate was 2 Hz. The external data storage was accomplished on the one hand via Ethernet to the central measuring computer and on the other hand, data records were sent via the global system for mobile communications (GSM) worldwide to a selected recipient even in poor reception conditions. Due to the redundant data acquisition and accurate recording of the cell’s operating history for a status analysis, the state of health could be precisely reconstituted so that the battery cells could be potentially transferred to a third life cycle in later use. For a better understanding of the configuration and functioning, a circuit diagram of the battery management system is attached as Figure A2 of the Appendix A.

#### 4.2.6. Auxiliary Equipment

Since increased safety concerns prevail for second-life batteries due to their often unknown pre-aging background, additional state monitoring sensors are beneficial for the operation. For this reason, the first string of the energy storage (see left battery block in Figure 1) was equipped with fiber-optical sensors that measured the temperature profile of the cell current collector redundantly to the resistant-electrical sensors of the battery management system. The main advantages of this method are the integrability of all twelve required sensor positions in a single measuring line and the immunity to electromagnetic fields occurring at high current change rates. If a defined limit temperature is exceeded, an alert notification is sent to the battery management system as the higher-level monitoring device, so that the relevant string with the detected malfunction is switched off.

To supplementary protect the battery cells from fault current spikes and overload, each string was provided with a 100 A fuse. The strings were combined via a busbar, which in turn was equipped with a 250 A fuse (simplified illustration in the middle of Figure 1). The merged battery current flows through a relay, which could be controlled by the master battery management system to directly disconnect the circuit in the event of a fault. In addition, the management system was linked to the off-grid converter via Ethernet to transmit the present battery state of charge, state of health and selected temperatures as well as any other occurring errors. If the state of charge falls below 15%, the off-grid inverter disconnects the external AC grid; from 5%, the internal grid, for recording the measured data, was also switched off. Once again, by exceeding 25% by charging, the external power network was activated.

In addition to the direct link between the battery management system and the converter, inside the battery powerhouse, an Ethernet-based local area network existed, which connected the central measuring computer with the converter as well as the two monitoring units, battery management system and fiber-optical sensor device. The measurement data of the individual components stored on the hard disk of the computer could be accessed remotely via a network router with a universal mobile telecommunications system (UMTS). For this type of data transmission, however, a sufficient mobile radio standard must be available. Since there was no, or only an unreliable, UMTS connection at the location of the energy system on Kibumba Island, the independent state value transmission function of the battery management system was of particular importance. Besides the radio antenna, a solar meter (SPM1, PCE, Meschede, Germany) for the measurements of the solar radiation power, which was used for the system design and efficiency determination, was also connected to the central measuring computer. 

For a high-performance operation of the system with low losses due to heat generation or fault currents, suitable and generously dimensioned cables were a deciding factor. In order to minimize these losses, all electric cable types and diameters as well as screw connections and contact pressures were selected in accordance with the directive DIN VDE 0276-604. Furthermore, system components were arranged in such a way that only the shortest possible cable lengths were required. This was particularly important for the DC transmission between the photovoltaic installation and the off-grid converter. In the next section, necessary work steps and required expenses for the system built-up process were described.

### 4.3. System Setup Procedure

The construction and implementation of such a plant in the prototype stage were very complex and a multi-faceted endeavor that could not be entirely captured in this section. This particularly concerned the development, work and travel effort of the employees involved in the project. Although that component undoubtedly represented the greatest expense, it had to be assessed individually as a result of the specific circumstances and was consequently not considered in the following. Devices available on the market, such as an off-grid converter (2750 €), the two DC/AC inverters (each 1100 €) and photovoltaic modules, could be simply accounted for. Since the latter were pre-used modules in the present case, no residual value could be specified; new solar components of these performance class would cost approximately 3500 €. An indication of the value of the battery was problematic because during the use as a vehicle traction battery it was already a prototype. The current target cost of second-life battery storage was given in the introduction as 88 €/kWh. With the existing battery capacity, this equated to a price of 7000 €. In addition, there were costs for electronic small parts and power cables as well as for the individual frame of the photovoltaic system of 1500 €, so that the total value of the system located on Kibumba amounted to approximately 17,000 €. In the following Table 2, the total expenses of the setup were composed. Besides transportation issues, various permits and customs documents were required. Personnel costs were not addressed.

The sole weight of the cells was 670 kg, the total for the battery was 1350 kg and with included packaging as well as additional components it was 1500 kg. Furthermore, the solar panels together with the foundation (900 kg), fixing material and power cables, especially those between photovoltaic system and inverter (400 kg) as well as tools and equipment (200 kg) resulting in a total freight weight of 3000 kg. Especially the reloading of the goods from the container to the truck, from this to the transport boat and from the landing place to the location of the system setup were not to be underestimated logistical challenges. In this specific case, as a result of a total lack of technical support, up to eight casual workers were employed at the various sites with a daily wage of 4 €/person. 

The focus of this pilot plant was on the demonstration of the technical and organizational feasibility as well as the acquisition of research data. On the basis of the knowledge gained, the derived blueprint would offer the possibility to carry out future, more economically oriented projects efficiently. Savings potentials for these projects constituted economies of scale in logistics processes and cost advantages in purchasing, most notably for pre-used batteries, and transporting large quantities of the equipment in general. Furthermore, the installation by trained local personnel following standardized process steps could be more cost-efficient leaving only the commissioning to the system engineers. Finally, the use of the specially designed resilient battery management system and outlined installation tailored to tropical or remote regions represents a major saving for future projects. After the setup and commissioning of the energy system, various experiments were carried out on the performance of the components. In the following Section 5, these tests were discussed.

## 5. Monitoring and Technical System Validation

**Figure 2:**
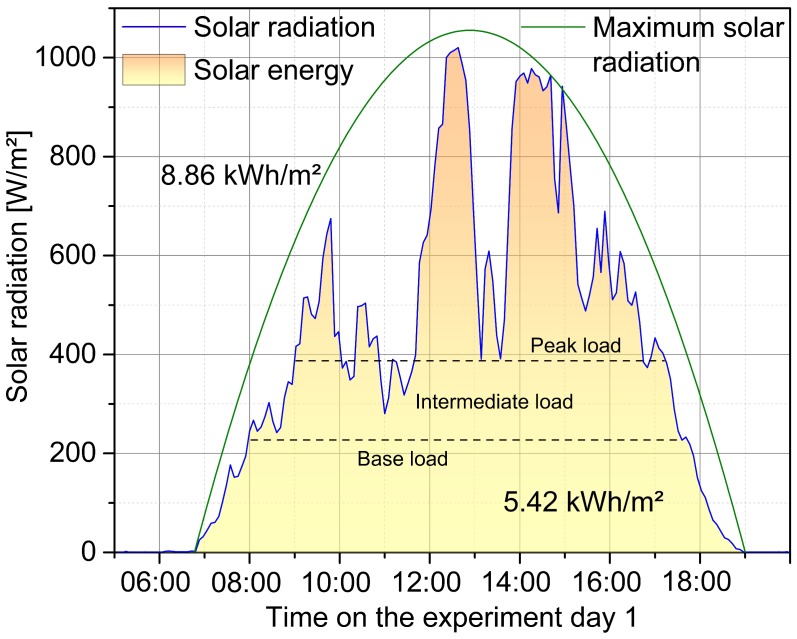
Measured, areal normalized solar radiation power at the site of the plant. The highest recorded value was 1020 W/m^2^. Over the entire day considered, with a total sunshine duration of approximately twelve hours, the solar radiation energy was 5.42 kWh/m^2^. Three weather- and daytime-dependent zones could be identified. The base load up to a power of 225 W/m^2^ was available over a time period of ten hours. The intermediate load up to a power of 390 W/m^2^ was available for about eight hours and the exceeding power was allocated to the peak load. As the maximum daily energy input under optimum conditions, a value of 8.86 kWh/m^2^ was approximated by the green parabola.

The aim of the investigation was to examine the efficiency and reliability of the system as well as to validate the previously made assumptions and performed measurements. The testing presented in the following takes place within a period of two days. The battery storage was shipped to Tanzania with a state of charge of 33%, which decreased until the finalization of the plant through consumption during assembling to approximately 26%. On the first day, the battery was charged throughout the entire sunshine duration with photovoltaic power; meanwhile, the state of charge increased to 80%. At the same time, the radiation power prevailing at the solar modules was continuously measured utilizing the solar power meter. After sunset, three halogen lamps, each with a rated power of 1.15 kW, were connected to the system as consumers. With sunrise the next day, the consumers were completely turned off. Subsequently, the recharge of the battery storage was monitored for several hours before the measurement was terminated. The data obtained from the initial measurement of the solar irradiation energy are graphically represented in Figure 2.

The measured total energy on experiment day 1 of 5.42 kWh/m^2^ was in good accordance to the pre-measurements, which were taken as the basis for the system design. Taking into account the effective solar area of 64.25 m^2^ as well as the efficiencies of the modules of 13.5% and the system of 90%, feed-in energy of 42.31 kWh was available. At a maximum approximated solar energy of 8.86 kWh/m^2^, up to 69.16 kWh could be fed into the system, which was 1.63 times the actual measured value. During periods without connected loads, the maximum storable power was limited by the off-grid converter’s rated charging current of 115 A, which corresponded to a power of 5.11 kW at a standard battery voltage of 44.4 V. Including the system parameters explained above, this limitation occurred with a solar radiation output of 654 W/m^2^. According to the rated power of the converter and based on the maximum battery voltage of 50.4 V, up to 5.80 kW could be processed for a short time with a converter’s maximum temporal charging current of 140 A even 7.06 kW, which corresponded to a solar radiation of 743 W/m^2^ and 904 W/m^2^, respectively. The latter value fitted well with the measured solar radiation profile of 99% or 41.92 kWh of the total energy of the sun, theoretically convertible by the system, could be transformed into a usable form. Based on the rated charging current of the converter and the standard battery voltage, 90% or 38.14 kWh could be stored by the system without any connected loads. When electricity was consumed in addition, the two DC/AC inverter fed directly into the stand-alone grid with a total rated power of 10.00 kW, so that, if the grid system was sufficiently efficient, the entire solar energy could be transmitted, also in the case of an already fully charged battery.

**Figure 3:**
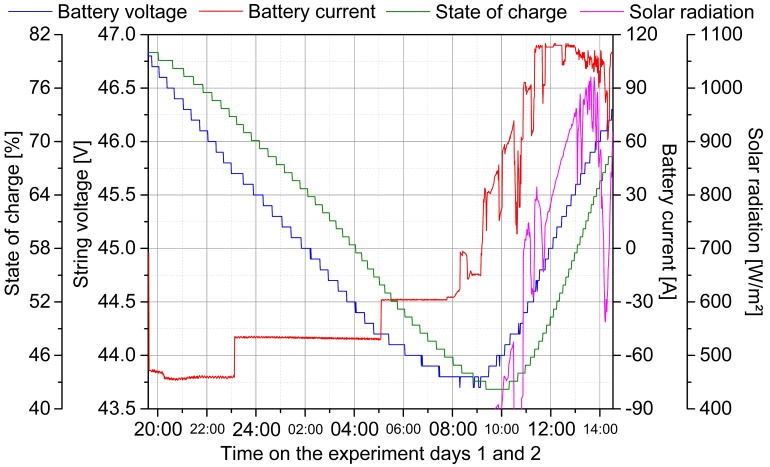
Charge and discharge experiment for the validation of the system performance and parameters. Plotted are the battery values voltage, current and state of charge as well as the solar radiation. At sunset on the first day of the experiment at 19:30, a defined load is connected to the system until the following day at 08:30, reducing the state of charge from 80% to 42% and the voltage from 46.6 V to 43.8 V. At 09:15 the DC/AC inverters are switched on to determine a starting point for the power feed. In the considered period until 14:30, the state of charge rises again to 68% and the battery voltage to 46.3 V. First at 11:20, the maximum charging current is reached for the first time.

Distinctive in the measured solar radiation profile were the strong power gradients due to shading. Even during the period of maximum insolation, the solar radiation dropped into the intermediate load range, which was classified up to 390 W/m^2^ as shown in Figure 2. Without the peak load range, an energy potential of 3.71 kWh/m^2^ was available. The base load range, up to a set power level of 225 W/m^2^, covered a daily capacity of the energy of 2.35 kWh/m^2^. After completion of the solar measurements, the DC/AC inverters were switched off and controlled discharge of the battery was initiated. The logged measuring data during this process are shown in Figure 3.

In order to preferably carry out the discharge experiment in the linear nominal voltage range of the battery, an initial state of charge of 80% was appropriate. After switching on the three bulbs as a load, a current of 73 A was provided by the battery, which corresponded to a power of 3.40 kW. At 23:10 there was a failure of one lamp, so that the current dropped to 50 A, and thus, with the preceding battery voltage of 45.7 V and a state of charge of 73%, a power of 2.29 kW was delivered. On experiment day 2 at 05:10 the second lamp failed at a state of charge of 54%. As a result, at a voltage level of 44.2 V, the current supply decreased to 29 A with a remaining power of 1.28 kW. By the end of the discharge experiment at 08:15, a total energy of 29.03 kWh was removed from the battery with an average power of 2.29 kW over a period of twelve hours and 40 min. Meanwhile, the state of charge dropped from 80% to 42% by 38 percentage points. Based on the discharged energy and the variation in the state of charge, an actual battery energy content of 76.39 kWh results, which was 4% less than assumed for the system design. Possible reasons for this were internal losses, self-consumption of the monitoring or progressive aging during the transportation due to unfavorable ambient conditions.

**Figure A3:**
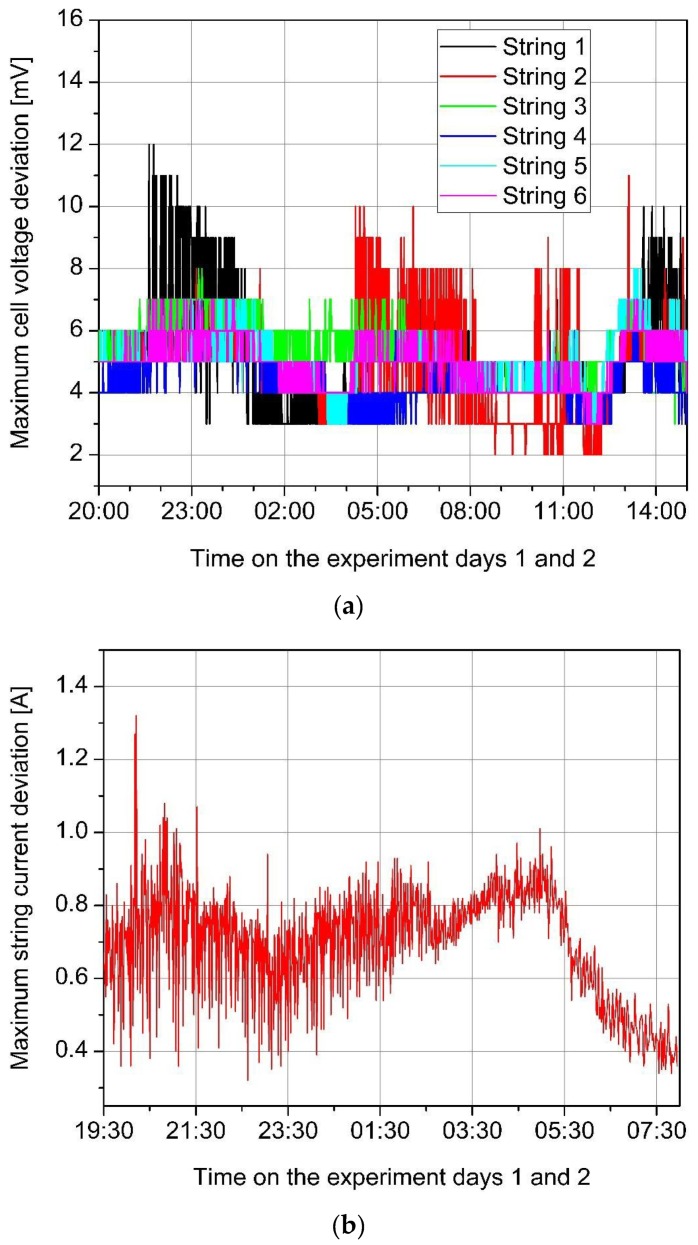
Maximum cell voltage deviation and string current deviation during the discharging and charging experiment presented in Section 5: (**a**) The differences of single cell voltages in a serial connection within the individual strings, which should be monitored in a second-life lithium ion battery operation in order to indicate possible mismatches due to progressive aging, are plotted in a selected time period; (**b**) the same applies to the differences of string currents within the six parallel connections of the module configuration.

The second part of the experiment started at 09:15 of the second day with the activation of the DC/AC inverters. The battery charging current varied significantly with the fluctuations of the solar radiation. Over a period of five hours and 20 min, the battery storage was supplied with an energy of 20.11 kWh, whereby the state of charge increased from 42% to 68%; this corresponded to a theoretical storage energy content of 77.36 kWh. The overall efficiency of the installed power system depended on solar radiation, converter operating mode, battery state of charge and ambient temperature. Hitherto, the efficiency of the system in charging operation mode was assumed to be 12.2%, composed of 86.5% loss on the solar modules and 10.0% loss due to conversion. For the analysis, exemplarily three characteristic operating points throughout the second experiment day were selected to approximate the actual efficiency. In order to demonstrate the system behavior in a realistic application scenario, the ice cube machine installed in the fishery was started at 08:35 in continuous operation as a base load with a DC-side power consumption of 0.68 kW. The first operation point considered was at 10:30 at a solar radiation of 520 W/m^2^. With regards to the effective solar module area, this corresponded to a total radiation capability of 33.41 kW. At this measuring point, a battery voltage of 44.2 V and a current of 72 A occurred, resulting in a charging power of 3.17 kW. Together with the 0.68 kW base load, there was a power supply by the system of 3.84 kW from the solar feed, which resulted in an efficiency of 11.5%. At 11:00 at 699 W/m^2^ with a power capability of 44.91 kW, the second operation point was defined. The battery had a voltage of 44.4 V and a charging current of 93 A at this moment. The power feed was 4.14 kW for the battery and 0.68 kW for the base load inside the grid, corresponding with an overall system efficiency of 10.7%. The last considered operation point was at 11:20 at a solar radiation of 767 W/m^2^ with a capability of 49.28 kW. At this moment, for the first time, the battery charging current reached its maximum with 115 A at a voltage level of 44.6 V and a power of 5.13 kW. Taking into account the prevailing base load of 0.68 kW, a total efficiency of 11.8% resulted. In this consideration, no internal losses of the battery were assumed because lithium ion battery cells generally had a very high degree of efficiency. For verification, based on the experiment depicted in Figure 3, the discharged energy quantity was compared with the charged energy quantity. In the largely linear voltage range from 45.0 V to 45.5 V, during the discharging phase 4.35 kWh was consumed to lower the voltage level by 0.5 V and during the charging phase 4.43 kWh fed to increase the voltage level by 0.5 V, resulting in a battery storage efficiency of 98.2%, justifying the previous assumption. However, with further aging, a decrease of efficiency could be expected and should be reevaluated. This degradation process was accompanied by a divergence of the states of health of the individual cells. For second-life batteries, single cell monitoring was therefore indispensable and consequently implemented in the developed battery management system. The corresponding measurement revealing the deviations of the respective individual cell voltages and string currents during the discharging and charging experiment is shown in Figure A3 of Appendix A.

**Figure 4:**
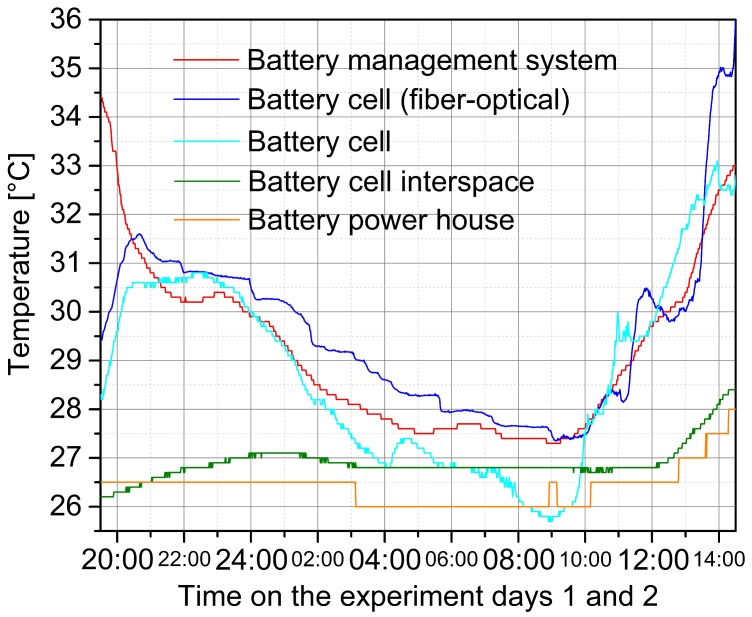
Characteristic temperature courses during the charge and discharge experiment. In addition to the temperature variation of the master battery management system, the values of an interspace of two centrally located cells within the first string are plotted. Furthermore, the temperature of the current collector of one of the last-mentioned cells is shown from the conventional resistant-electrical as well as fiber-optical measurement, whose setup is illustrated in Figure 1. For comparison, the temperature inside the battery powerhouse is additionally plotted. Due to the lower impact on safety and status analysis, this value is recorded with a lower resolution of 0.5 K.

While the second determined efficiency deviated perceptibly, the other two outcomes were in good accordance with the initial assumptions, so that the previous considerations of the implemented energy system’s processable solar radiation power were applicable. A certain degree of additional losses had to be expected since self-consumption of controlling components, active device cooling, system monitoring programs and cable resistances were not included in the valuation. The plant did not show limitations in performance and component alignment and thus met the imposed requirements. Furthermore, no abnormalities, faults or other problems could be located. Conclusively, an examination of selected temperature courses during the discharge and charge experiment was carried out. The respective measuring data are plotted in Figure 4.

The battery powerhouse was built of solid stone to achieve the lowest possible temperature fluctuations over the course of the day. The ambient temperature of the system was 26.5 °C at the beginning of the discharge experiment at 19:30. At 03:00 the temperature dropped to 26 °C according to the lower nighttime temperatures and temporarily rose to 26.5 °C at 09:00 due to activities in the house. Starting at 10:15, the value continued to increase, initially stable to 26.5 °C and from 12:45 more clearly in a short step sequence up to 28.0 °C until 14:30. A similar course was shown by the cell interspace temperature. Due to the cell activity in the supply of electricity, the temperature rose from the initial 26.2 °C to 27.1 °C at 01:00. Due to the dropping ambient temperature and the lower power demand from 23:00 through the failure of one load, again the value decreased slightly to 26.8 °C. From 12:00, the cell interspace temperature rose significantly to 28.5 °C, because on the one hand, with high power, the photovoltaic current was stored, and on the other hand, the ambient temperature increased accordingly.

The cell’s current collector temperature is one of the crucial state values for the battery management system calculations. For enhanced safety, which is a particular requirement of second-life batteries, the cells were equipped with both typical resistant-electrical and fiber-optical sensors for temperature monitoring. Caused by the high discharge rate at the beginning of the experiment, the cell temperature rose significantly from 28.3 °C to 30.8 °C (electrical sensor) or from 29.4 °C to 31.6 °C (optical sensor), respectively. Subsequently, with the decrease of the ambient temperature, a drop was detected, which accelerated after 23:00. At 09:00 the minimum temperature of 25.5 °C (electrical sensor) or 27.4 °C (optical sensor) was reached. It was noticeable that the negative temperature trend recorded to this point was markedly more pronounced in the case of the electrical sensor and had less continuity with an additional temporary maximum at 04:30. After 09:00, with the exception of a temporary maximum at 11:00, the value recorded by the electrical sensor increased largely constantly until 14:30 to 33.0 °C. Over the same period, the data acquisition of the optical sensor indicated a similarly rising cell temperature. At 11:20, when the maximum charging current was reached for the first time, the optically detected temperature increased markedly from 28.2 °C to 30.5 °C. After a temporary drop to 29.8 °C, the temperature rose again significantly to 35.8 °C at 14:30, which was a noticeably higher value compared to the electrical sensor. One reason for this could be the sunrays entering the house through ventilation slits, which have a different strong effect on the measuring principles.

Finally, the internal temperature profile of the battery management system was examined. With the gradual termination of the charging experiment on day 1 at 18:00, the device went into balancing mode. Due to charge balancing, ohmic losses occurred so that the temperature at the main monitoring device increased. With the start of the discharge, the balancing operation ended, which is why the measured temperature initially dropped from 34.5 °C to 30.2 °C. Due to a higher system temperature during discharging, at 23:00 a temperature increase of the management system to 30.5 °C was measured. Subsequently, the temperature dropped overnight to 27.5 °C and increased again with the start of charging. Meanwhile, with the failure of the second lamp at 05:00 on the second day of the experiment, a short-term rise in temperature by about 0.5 K could be detected. From 13:00 a much stronger increase occurred, ending at 14:30 at a temperature of 33.0 °C.

Despite the high load of the battery storage unit under high ambient temperatures, no abnormalities in the temperature measurement, neither on the cells nor in the monitoring electronics, could be determined. The maximum value recorded was 35.8 °C, which was within the system specifications of the battery and electronics, that ensured a safe operation up to 60 °C or 85 °C, respectively. Due to the low impedance of the battery, the rated current was 160 A per single string and thus 960 A for the entire storage, which was both in the charging and discharging mode that was by far more efficient than required by the system parameters. Batteries suffer thermal losses especially in the range of the charge and discharge cut-off voltage. In particular, lithium ion-based systems are most susceptible to degradation at very high or low state of charge levels. In the use as stationary energy storage, second-life lithium ion batteries should preferably be operated in a limited voltage range. After the termination of the test series, the cell threshold voltage values were set to 3.2 V and 4.0 V to achieve a prolonged second life cycle. As consumer demands increase, the voltage boundaries can be reset.

Based on the peak-load tests carried out, it was shown that the installed energy system, on the one hand, meets the electrical performance requirements, and on the other hand, did not exceed any specification limits during operation. It was also demonstrated that the design and the dimensioning of the components were consistent as well as that assumptions about efficiencies and states of health were correct. Since no realistic consumer profiles due to the deferring connection of loads were available up until now, no appropriate estimation of the expected service life could be obtained. In the following Section 6, a holistic assessment of the project, not primarily in terms of technical validation, but rather in a resource-related and socio-economic manner, was executed.

## 6. Regional Development Impact, Sustainability and Future Potential

The government of a country has the fundamental task of ensuring the best possible development opportunities in its own territory. Since the availability of electricity is a basic prerequisite for this, in most cases a national supply utility company exists, which is responsible for the overall connectivity and public power provision. With limited available governmental budget resources, those areas to be electrically supplied will be explored first and foremost, which are located close to already installed infrastructure and thus are easily accessible; this procedure represents the first implementation scenario in electrification. Rural regions, further away from the national grid, may be economically attractive to access with electrical infrastructure if there is a clear economic potential that can develop after connection. This second implementation scenario requires specialized organizations that hold the necessary know-how for the occurring characteristic circumstances. Furthermore, there are areas that are both remote and lack economic potential. Despite this, under certain preconditions, it is feasible to carry out electrification, as a high humanitarian impact would prevail. For this third implementation scenario, the applied key decision variables must be supplemented by non-economic factors, so that a power grid connection can still be assessed positively if specific location factors, such as health care, education, nutrition or business start-up opportunities appear. As demonstrated by the concrete case of the island of Kibumba, the existence of a school and an infirmary with catchment areas extending beyond the territory of the island itself, as well as the potential to establish a commercial fishery led to a positive evaluation under the terms of the diversified key decision variables, although economic location factors did not sufficiently prevail.

Already the impact of the availability of electrical light was a central point in society’s development possibilities since private and business activities were no longer limited by daylight hours and also safety-related issues could be overcome. The access to internet and the use of computers, facilitated by electricity, significantly increased the educational standards of the inhabitants. A future-oriented apprenticeship of the students was an essential contribution to equality of opportunity. In addition to education, a reliable health care system was the basic prerequisite for the emergence of a prosperous society. The first step that this project had initiated, was the possibility of cooling the most important medicines and lighting for a 24-h physical examination. On the basis of this, medical equipment for an advanced treatment could now be procured. Although, surprisingly, it does not appear to be widespread regionally on Kibumba, malaria is the dominant disease throughout the lake and coastal areas. Additionally, bilharzia and otherwise contaminated water pose a major health risk for the inhabitants. Despite its location in the middle of a freshwater reservoir, the supply of drinking water, which is largely collected by roof drainage cisterns, remains a serious challenge. Accordingly, polluted and contaminated lake water is used for cooking and washing, which is particularly critical in the shore area due to the higher temperatures and the lower water change. The lack of sanitation is an added negative factor. Water can be treated thoroughly by boiling and filtering with an electrical appliance, which should be considered in the continued aid to this region. The third principal factor for livability is nutrition. The main supply is contributed due to the geographical location by fishing. This activity is moreover the only opportunity for economic development. The installed refrigeration system with icemaker, which allows the fish catch to be cooled overnight or for longer journeys to lucrative marketplaces without interruption of the freezing chain is therefore very important for this potential industry.

**Figure 5:**
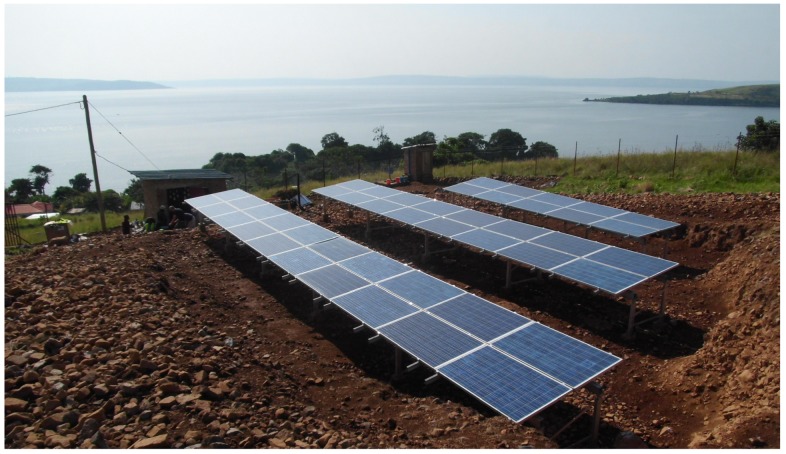
System overview picture with photovoltaic installation in the foreground and the battery powerhouse on the left. For the solar modules, the area was leveled out to achieve good alignment. The visible power pole represents the connection point to the external stand-alone grid. For security reasons, the property is fenced in. This kind of project in the vicinity of water bodies also requires sanitary facilities, which can be recognized in the center of the picture. In the distant background with a view to the west, the mainland is already noticeable.

As discussed above, the energy system that was assembled within the framework of this project, together with the additional equipment procured in the parallel sub-projects, generated a clear and multifaceted humanitarian impact. In Figure 5, an overview photograph of the final system structure is shown. In its technology level, it represents a clear contrast to the surroundings. This was exactly one of the main challenges. While energy systems with lead-acid batteries rely on an established, robust and comparatively simple technology, systems based on lithium ion batteries consist of highly complex components that can only be handled by specialists. For the sustainable development of a defined region, it is desirable that the available technology leads to an increase in the knowledge of the population. To achieve this, residents must be involved in system planning and implementation already from the outset. The operating principles should be understood in order to react appropriately in the event of a fault or incident; this also includes information on the dangers of handling the electric current. The lack of knowledge transfer clearly marks an essential point of criticism in the self-reflection of this project. The basic fears of contact and language barriers must be overcome through close involvement of local authorities, which has to be considered during the development of comparable projects. In any case, it is, nevertheless, necessary to attach the greatest importance to the longevity of the overall system, especially for plants in rural regions. The implementation of the energy system on Kibumba was therefore particularly focused on this.

Due to the deliberately robust design of the electronic components, the system was not expected to fail abruptly but degrade gradually. A period of ten years was specified as the minimum service life. The commercially purchased components such as the off-grid converter and the DC/AC inverters meet this requirement according to general experience. The solar modules aged by 10% during their first decade of existence. It was assumed that the linear degradation of 1% per year will continue at a constant level so that after ten years an efficiency of 80% would remain, which corresponded to a remaining daily feed-in capacity of 33.85 kWh taking into account the further previously determined system efficiencies. For the battery, a prognosis is very difficult, because the demand profile had completely changed compared to the first life cycle. However, the cells had lost 7% capacity in four years, which corresponded to an annual rate of 1.75%. As a result, the battery capacity would still be 64.18 kWh after ten more years of service with a constantly progressing degradation. There was no forecast for the consumer profile after the next decade, which was why it was presumed that the consumption anticipated during the system design of 18.75 kWh would be steady after five years. Unlike in the automotive industry, no conclusive storage capacity wear limit could be specified for stand-alone power systems, as it is largely dependent not only on customer behavior but also on the type of supply contracts, customer flexibility and structure as well as the weather situation. From the installer’s point of view, the system is no longer sufficiently efficient as soon as the electricity requirement can no longer be covered for one daylight and two night periods without additional solar supply. Over these 36 h, consumption of 28.13 kWh is expected. Based on the aging process described above, the feed-in capacity of the solar modules would drop to this value after a further 30 years of operation in the system. By then, the battery would still have an energy storage capacity of 40.38 kWh. Nevertheless, a lifetime prognosis of more than ten years is theoretical due to the complexity of the installation. 

Of great relevance is the disposal of the individual components, especially the photovoltaic panels and the lithium ion battery. As pointed out, in a stand-alone grid even electricity from second-life batteries was virtually unaffordable for the average citizen in the area under consideration. However, there are multiple application possibilities without significant power demands, which would already contribute substantially as improvements in everyday life, for example through LED lighting or mobile phone charging. After the end of its current use, the second-life battery system can therefore be transferred into several individual systems in their third life cycle; the necessary parameter settings and operating modes are already integrated into the developed battery management system presented here. Irrespective of the possible continued use, the disposal case will occur, and in all circumstances, it must be prevented that components of the battery get in contact with the ground or lake water. It is essential, for this reason, to hold the battery cells in the possession of a responsible organization and to lend them exclusively to private individuals by means of a deposit system. The disposal, and in the preferable case, the recycling of the lithium ion battery is a potentially developing industry due to containing raw materials. Generally, the disposal of discarded objects on the African continent is considered critical. All the more important is the specification of governmental framework conditions, which have to be enforced accordingly. In the case of the pilot project, the electricity supplier and operator of the energy system had assured that the batteries would be recycled by certified companies when their useful life ends. This is also an important prerequisite for future projects in order to ensure a sustainable and safe goods cycle. On the northern shore of Lake Victoria, in the Ugandan area around Kampala, several companies, e.g., Asante Waste Management Ltd. and Bodawerk International Ltd., already exist that have specialized in the recycling of batteries. Furthermore, clear regulations for storage and disposal exist, so that good cooperation between battery power suppliers and waste disposal companies in geographical proximity can be established on this basis.

For the research institutions involved, this project is about the outline of a blueprint as well as the implementation of a pilot plant, which is not exclusively seen as a contribution to the development of a remote region, but also as an establishment of a quantifiable expertise and the creation of practical test opportunities for second-life batteries in tropical environments in a stand-alone power network operation. The technical and organizational knowledge gained in this way can be directly incorporated into the process efficiency in the realization of future projects, thus significantly improving their cost–benefit ratio. For example, in Lake Victoria alone there are numerous islands with parameters similar to those of Kibumba. Through less cumbersome adaptation without entirely newly engineered solutions and organizational pioneering work, a system implementation can be beneficial even from an economic point of view. The cost factors listed in Table 2 must therefore be supplemented by the personnel required for component manufacture or selection, land consolidation, maintenance and disposal. In addition, the expenditure on cabling has to be considered for the respective area. The total amount is then depreciated over a calculation period of, for instance, ten years, whereby at least the loss in value must be covered annually by the customers. A detailed profitability analysis will be the content of a subsequent publication in which, moreover, the socio-economic aspect will be in focus.

## Figures and Tables

**Table 1:** Examples of projects for stationary energy storage with vehicle lithium ion batteries after their first life cycle.

Project Name	Capacity	Partners	Location
E-mobility thought through to the end [23]	13.0 MWh	Daimler, The Mobility House, GETEC, REMONDIS	Lünen, Germany
ESS at Johan Cruijff ArenA [24]	3.0 MWh	Nissan, Eaton, BAM, The Mobility House, Johan Cruijff ArenA	Amsterdam, Netherlands
Battery 2nd Life [25]	2.8 MWh	Bosch, BMW, Vattenfall	Hamburg, Germany
Second-Life Grid-Tied Storage Program for EV Battery Packs [26]	400 kWh	Sumitomo, Nissan, 4R Energy, Green Charge Networks	Yumeshima (Osaka), Japan
Energy Local Storage Advanced system (ELSA) [27]	11–88 kWh	ASM, AÜW, B.A.U.M, Bouygues, Nissan, Renault, RWTH Aachen	Six pilot sites in France, Germany and Italy

**Table 2:** Listing of the system implementation expenses in terms of work or organization packages, costs and duration.

Implementation Step	Expenditure	Time Exposure
Approval for transportation of dangerous goods (Germany)	550 €	Obtained in advance; procurement period about two weeks
Loading and truck transport from Goslar to Hamburg	950 €	Five hours
Customs inspection for export	275 €	Time delay about one day
20-foot container shipment from Hamburg to Dar es Salaam	1325 €	Approximately four weeks
Pre-shipment verification (replacement for import customs inspection)	225 €	Waiting time in the import customs area uncertain, in this case one week
Truck and boat transportation from Dar es Salaam to Kibumba	500 €	One week
Assembly, commissioning and testing of the energy system	17,000 € without staff	Four days assembly, two days testing (conducted with three engineers)

## Appendix A

The Appendix A contains Figure A1: Basic module configuration, Figure A2: Circuit of the battery management system and Figure A3: Cell voltage and string current deviations.
